# On the Resistance Coefficients for Heat Conduction in Anisotropic Bodies at the Limit of Linear Extended Thermodynamics

**DOI:** 10.3390/e27030314

**Published:** 2025-03-18

**Authors:** Devyani Thapliyal, Raj Kumar Arya, Dimitris S. Achilias, George D. Verros

**Affiliations:** 1 Department of Chemical Engineering, Dr. B.R. Ambedkar National Institute of Technology, Jalandhar 144011, Punjab, India; devyanithapliyal5@gmail.com (D.T.); or rajaryache@gmail.com (R.K.A.); 2 Department of Chemistry, Aristotle University of Thessaloniki, 54124 Thessaloniki, Greece; axilias@chem.auth.gr

**Keywords:** heat conduction, extended thermodynamics, anisotropic body, crystals, Onsager reciprocal relations

## Abstract

This study examines the thermal conduction resistance in anisotropic bodies using linear extended irreversible thermodynamics. The fulfilment of the Onsager Reciprocal Relations in anisotropic bodies, such as crystals, has been demonstrated. This fulfilment is achieved by incorporating Newton’s heat transfer coefficients into the calculation of the entropy production rate. Furthermore, a basic principle for the transport of heat, similar to the Onsager–Fuoss formalism for the multicomponent diffusion at a constant temperature, was established. This work has the potential to be applied not just in the field of material science, but also to enhance our understanding of heat conduction in crystals. A novel formalism for heat transfer analogous to Onsager–Fuoss model for multicomponent diffusion was developed. It is believed that this work could be applied for educational purposes.

## 1. Introduction

Over the past few decades, irreversible thermodynamics has emerged as a potent method for the derivation of macroscopic laws of processes with its application both to the Euclidean framework and to relativistic spacetime [1,2,3]. It is worth mentioning that from the inception of irreversible thermodynamics, extensive study has been performed on its axiomatic system, which is founded on axioms that go considerably beyond those of equilibrium thermodynamics.

The Onsager reciprocal relations (ORR) are fundamental principles in irreversible thermodynamics. ORR states that the matrix of phenomenological resistance coefficients (R) in the flux-force relations is symmetric (R_ij_ = R_ji_) when there are no magnetic fields and when the fluxes J or thermodynamic forces X are linearly independent (Linearity Axiom: **X = R.J**) [4,5,6]. Miller [7,8] presented experimental evidence of ORR, while Truesdell and colleagues [9,10] expressed significant scepticism regarding the theoretical basis of ORR. Onsager [4,5] employed the concept of microscopic reversibility to derive these reciprocal relations; however, Truessdell and his colleagues [9,10] argued that the concept of microscopic reversibility is not consistent with Poincare’s recurrence theorem.

Today, ORR is widely recognized by most scientists in the area as being well-established, particularly in the context of irreversible thermodynamics that is near equilibrium [11,12,13,14,15,16,17,18,19,20,21,22,23,24]. Linear Extended Irreversible Thermodynamics (LEIT) is a reliable approach used to deduce fundamental principles governing macroscopic processes that occur far from local equilibrium. This method has significant practical uses in various materials. The ORR is regarded as an axiom in this region [14].

Heat conduction in anisotropic bodies, such as crystals, is closely connected to ORR. The creation of ORR near to equilibrium [4,5] was primarily motivated by the research conducted by Voigt and Curie on heat transmission in crystals [16,23]. The application of LEIT in the field of heat transmission is justified by assuming a sinusoidal starting disturbance of temperature at a specific place on an isolated body [14]. In this scenario, the Fourier law incorporates relaxation time factors as anticipated by the Linear Extended Irreversible Thermodynamics (LEIT) to prevent the violation of the second thermodynamic postulate caused by oscillations in the total entropy of the system [14]. The invention of the Cattaneo equation for heat transmission was motivated, in part, by this observation [14]. The development of Linear Extended Thermodynamics aimed to derive the Cattaneo equation by applying concepts of sound non-equilibrium thermodynamics [14].

Heat transfer in crystals is a significant factor in material science applications, including the improvement of heat transfer through the use of nano-material suspensions [25,26], as well as in the field of optoelectronics [27,28,29]. For instance, materials that are extremely thin at the atomic level, known as Van der Waals (vdW) materials, along with their related heterostructures, offer a versatile platform for manipulating heat transmission at the nanoscale and developing innovative thermoelectric materials [27,28,29].

Specifically, the study of heat conduction in crystals has received significant attention in recent years. Olson et al. [30] created models to establish a connection between the variable spatial anisotropy of heat conductivity and anisotropic crystal formations. Chen and Liu [31] designed a nonlocal discrete model to study anisotropic heat conduction phenomena and investigate the connection between bond properties and material thermal conductivity. In their research, Dong et al. [32] utilized modern computational techniques to investigate the heat transport and associated characteristics of a new crystal known as quasi-hexagonal-phase fullerene (QHPF). Their findings demonstrated that the thermal conductivity in QHPF exhibits anisotropy, making it a viable material for 2D electronic devices. Zhao et al. [33] investigated the thermal conductivity of a significant material at elevated pressure, with potential industrial applications such as diamond manufacturing. It has been demonstrated that the diamond displays clear anisotropic characteristics under a pressure of 120 GPa. Other important applications of heat conduction in crystals include the protein crystal growth rate by means of temperature [34] and thermodiffusion in protein aggregations [35]. Other materials exhibiting anisotropic heat transfer include the wood and the composite materials.

In our earlier study, we investigated the fundamental principles of Onsager Reciprocal Relations (ORR) in an isotropic fluid with isothermal multi-component diffusion, as described in the LEIT [36]. In addition, the Onsager–Fuoss formalism, and the Maxwell-Stefan model, which are basic laws governing isothermal multi-component diffusion in the LEIT, were derived [36].

The recent advance in irreversible thermodynamics [37,38,39] imposes a re-examination of fundamental principles of LEIT such as the ORR. The objective of this study was to investigate the validity of the ORR approach for modelling heat conduction in an anisotropic body, specifically in the absence of elasticity, mass transfer, or chemical reactions, at the limit of Linear Extended Irreversible Thermodynamics (LEIT) that is far from the local equilibrium process. Please notice that in this area ORR is regarded as an axiom [14]. Additionally, the aim was to establish a fundamental law for heat transfer, drawing an analogy to the Onsager–Fuoss formalism used for isothermal multi-component diffusion.

## 2. Theoretical Section

The entropy production rate per unit volume (*σ*) for heat conduction in an anisotropic body in the absence of mass transfer, elasticity, or chemical reactions is written as [14]:

(1) $$\sigma =\sum_{\iota =1}^{3}J_{\mathrm{qi}}\left(\frac{\partial }{\partial x_{i}}\left(\frac{1}{T}\right)\right)\delta_{i}=\sum_{\iota =1}^{3}J_{\mathrm{qi}}\left(-\frac{1}{T^{2}}\frac{\partial T}{\partial x_{i}}\right)\delta_{i}$$

where *x_i_* are the Cartesian coordinates, T is temperature and **δ_i_** is the unit vector in the ith direction. The following equation holds true by generalizing the Maxwell-Cattaneo equation [14] for heat conduction in an anisotropic body at the limit of LEIT:

(2) $$J_{qi}=-\sum_{j=1}^{3}\left(\tau_{ij}\frac{\partial J_{qj}}{\partial t}+K_{ij}\frac{\partial T}{\partial x_{j}}\delta_{i}\right)$$

The heat conductivity **K** is a second rank tensor in the case of anisotropic body.

Following Jou et al. [14] the entropy production rate per unit volume (*σ*) for heat conduction in an anisotropic body at the limit of LEIT might be derived immediately by fusing the Maxwell-Cattaneo law of heat conduction (Equation (2)) into Equation (1):

(3) $$\sigma =\sum_{i=1}^{3}J_{qi}\left(\frac{\partial }{\partial x_{i}}\left(\frac{1}{T}\right)\right)\delta_{i}=\sum_{i=1}^{3}\sum_{j=1}^{3}\left(\tau_{ij}\frac{\partial J_{qj}}{\partial t}+K_{ij}\frac{\partial T}{\partial x_{j}}\right)\left(\frac{1}{T^{2}}\frac{\partial T}{\partial x_{i}}\right)$$

The process of obtaining Equations (1)–(3) substantially adheres to the methods employed by Jou and colleagues [14].

In order to investigate the foundations of the ORR, the local heat transfer coefficients (**h_loc_**) close to the equilibrium (***τ*** = **0**) have been introduced [40]:

(4) $$-K_{ij}\frac{\partial T}{\partial x_{j}}=h_{loc,ij}(T-T_{0i})\;or-\frac{\partial T}{\partial x_{j}}=\frac{h_{loc,ij}}{K_{ij}}(T-T_{0i});\;i,\;j\;=\;1,\;2,\;3$$

where T_0i_ is a constant reference temperature located outside the heat transfer area at the ith axis. This procedure results in the replacement of the temperature gradient with the temperature difference. However, this is not a new or original idea; its inception can be attributed to reference [40]. Equation (4) indicates that the notion of heat transfer coefficients can be easily applied to heat transmission in solids. This observation aligns with established technical ideas, such as the film theory in heat transport [15]. In particular, film theory predicts: $h_{loc,ij}=\frac{K_{ij}}{L_{i}}$ where *L_i_* is the distance between the point where *T*_0__i_ is located, the point having temperature *T*. The above film theory prediction was recently generalized to heat transfer in microscopic level by Chen and Liu [31].

It is important to understand that thermal conductivity, represented by the tensor **K**, is a second-order tensor that describes heat transport in anisotropic media. In simpler terms, the temperature gradient in one direction impacts not only the heat flux in that same direction, but also in the other two remaining directions. Thus, it may be inferred that the local heat transfer coefficient tensor (**h**_loc_) is a tensor of second order.

A more profound question arises regarding the heat transfer coefficients that consider heat transfer occurring far from local equilibrium: How can one establish heat transfer coefficients for heat transfer far from local equilibrium (**τ** ≠ **0**) in a manner that also encompasses the scenario of heat transfer approaching equilibrium (**τ** = **0**)? In reference [40], the answer to this dilemma is provided by developing the idea of residual heat transfer coefficients (**h**_res_). In particular by combining the local heat transfer coefficient close to the equilibrium (**h_loc_**) with the residual heat transfer coefficient (**h**_res_) the heat flux in an anisotropic body at the LEIT is expressed as:

(5) $$J_{qi}=-\sum_{j=1}^{3}\left(\tau_{ij}\frac{\partial J_{qj}}{\partial t}+K_{ij}\frac{\partial T}{\partial x_{j}}\delta_{i}\right)=h_{res,i}\sum_{j=1}^{3}h_{loc,ij}(T-T_{0i})\delta_{i}=h_{i}\left(T-T_{0i}\right)\delta_{i}$$

where *h*_i_ is the overall heat transfer coefficient. The above law is Newton’s law of cooling or heating written in three-dimensional form [15] and it could be used as an ad hoc definition of both the residual and overall heat transfer coefficients. The concept of heat transfer coefficients is well established in the literature, and it is widely used in the designing of complex engineering applications [15] by using the film theory. This theory predicts: $h_{loc,ij}=K_{ij}/L_{i}$ [15]. A typical example is the heat transfer coefficient of a pipe wall participating in a heat transfer process. In engineering applications, we have to also consider in calculations the heat transfer coefficient of pipe wall. In film theory this coefficient is approximated as the ratio of wall thermal conductivity divided by wall thickness.

Let us now assume that the heat fluxes are linearly dependent. This is achieved by multiplying both left- and right-hand side of Equation (5) defining the overall heat transfer coefficients (Equation (5)) by a parameter **λ** and setting the summation equal to zero. Accordingly, the following relations could be directly derived:

(6) $$\begin{array}{c} \sum_{i=1}^{3}\lambda_{i}J_{qi}\delta_{i}=\sum_{i=1}^{3}\lambda_{i}h_{i}\left(T-T_{0i}\right)=0,\;\sum_{i=1}^{3}w_{i}=1\mathrm{or}\;\sum_{i=1}^{3}w_{i}T_{0i}=T;\;\\ \sum_{i=1}^{3}w_{i}J_{qi}\delta_{i}/h_{i}=0 \end{array}$$

where **w** = **λ**·**h**.

The above equations are analogous to the respecting equations of multi-component diffusion [36,41].

One can directly show by further using Equations (4) and (5) the following relationship between relaxation times (**τ**) and heat transfer coefficients:

(7) $$\sum_{j=1}^{3}\left(\tau_{ij}\frac{\partial J_{qj}}{\partial t}\right)=\left(1-h_{res,i}\right)\sum_{j=1}^{3}h_{loc,ij}(T-T_{0i})\delta_{i}$$

By introducing Equation (5) into Equation (3) one could write the entropy production rate σ in terms of the overall heat transfer coefficients as:

(8) $$\sigma =\sum_{i=1}^{3}\sum_{j=1}^{3}\left(\tau_{ij}\frac{\partial J_{qj}}{\partial t}+K_{ij}\frac{\partial T}{\partial x_{j}}\right)\left(\frac{1}{T^{2}}\frac{\partial T}{\partial x_{i}}\right)=\sum_{i=1}^{3}h_{i}\left(\frac{\partial T}{\partial x_{i}}\frac{1}{T^{2}}\right)(T-T_{0i})$$

After introduction of the transformation $T_{0i}\to T_{0i}+T_{0}$ to the above equation, the following equation has been directly derived by assuming that the entropy production rate σ is uniquely defined and independent of the constant temperature T_0_:

(9) $$\sum_{i=1}^{3}h_{i}\left(\frac{\partial T}{\partial x_{i}}\frac{1}{T^{2}}\right)=0\mathrm{or}\;\sum_{j=1}^{3}h_{j}X_{j}=0$$

The above transformation implies that the reference temperature T_0i_ is changed by adding a constant temperature T_0_. A clear physical meaning for this transformation results by considering the heat transfer system on a vehicle moving with a constant and uniform velocity. Obviously, an observer on the ground will observe the particles of the heat transfer system moving with an additional velocity, corresponding to an additional temperature T_0_. However, our observations in Euclidean geometry indicate that the temperature of the system remains unaffected. X is the thermodynamic force defined as:

(10) $$X_{i}=\left(\frac{\partial T}{\partial x_{i}}\frac{1}{T^{2}}\right);\;i\;=\;1,\;2,\;3$$

The linearity postulate [42] is strongly related to the above transformation. By taking into account the above definition of thermodynamic forces and applying the linearity postulate to Equation (8) the following equation has been derived:

(11) $$X_{i}=\sum_{j=1}^{3}R_{ij}J_{qj}=\sum_{j=1}^{3}R_{ij}h_{j}(T-T_{0j});\;i\;=\;1,\;2,\;3$$

where R_ij_ are the resistance coefficients. One could anticipate based on the similarities between heat and mass transfer that resistance coefficients are functions of intensive variables of the system such as temperature.

By applying the transformation $T_{0i}\to T_{0i}+T_{0}$ to the above equation and again, by assuming that the driving thermodynamic force is independent of the constant temperature, T_0_, the following restrictions are directly derived:

(12) $$\sum_{j=1}^{3}R_{ij}h_{j}=0;\;i\;=\;1,\;2,\;3$$

The following is the result of applying the linearity principle to Equation (9):

(13) $$\begin{array}{c} \sum_{k=1}^{3}h_{k}X_{k}=\sum_{k=1}^{3}h_{k}\sum_{i=1}^{3}R_{ki}J_{qi}=\sum_{i=1}^{3}J_{qi}\sum_{k=1}^{3}R_{ki}h_{k}=0\;\mathrm{or}\;\\ \sum_{k=1}^{3}R_{ki}h_{k}=\sum_{j=1}^{3}R_{ji}h_{j}=0;\;i\;=\;1,\;2,\;3 \end{array}$$

The aforementioned equations are valid because the fluxes are specified in terms of an arbitrary temperature and in the most generic situation $\sum_{i=1}^{3}J_{qi}\neq 0$. The mathematical part of Equation (13) is due to Tyrrell and Harris [11].

According to Equations (12) and (13) above, the square matrix of R is symmetrical (R_ij_ = R_ji_), or in other words, the ORR holds true.

It is worth mentioning that the mathematical methodology applied in this work is not a novel idea; The above mathematical methodology was validated in previous work [41,43] for ORR in the case of isothermal multi-component diffusion [41] and simultaneous heat and mass transfer [43] close to equilibrium. Moreover, the mathematical methodology described in previous work [41,43] was recently generalized [37] for multi-component diffusion close to equilibrium. However, this work deals with heat transfer in LEIT framework that is far from local equilibrium process [14].

However, in previous work [36] fundamental equations such as the Onsager–Fuoss model for isothermal multi-component diffusion at the limit of LEIT were derived:

(14) $$X_{di}={\left(\nabla \mu_{i}\right)}_{T,P}+a_{di}\frac{\partial J_{i}^{*}}{\partial t}=-\sum_{j=1}^{n}c_{j}R_{d,ij}\left(v_{j}-v_{i}\right)$$

where *X*_di_ is the thermodynamic driving force for diffusion, *a*_di_ is a thermodynamic parameter, *c*_j_ stands for molar concentration of the j-th substance, *μ*_i_ is the chemical potential under constant temperature (T) and pressure (P), $J_{i}^{*}$ represents the molar flux of the i-th substance relative to the centre of mass, $v_{i}$ is the velocity of the i-th substance, *R*_d,ij_ is the resistance coefficient for diffusion between the i-th and the j-th substance, respectively.

The above equation could be viewed as an intermediate step to the derivation of Maxwell-Stefan equations that are directly obtained from Equation (14) by using the standard definition of chemical potential [36].

A deeper question arises from the preceding analysis: Does any parallel exist between the laws governing heat transfer and the fundamental laws governing diffusion, such as the Onsager-Fuoss model elucidated in Equation (14)?

Please, note that by applying the linearity postulate and the ORR to the dissipation function, multiplying Equation (12) by (T-T_0i_), and subtracting it from the linearity postulate Equation (11), one could arrive at the following equation:

(15) $$X_{i}=\left(\frac{\partial T}{\partial x_{i}}\frac{1}{T^{2}}\right)\delta_{i}=-\sum_{j=1}^{3}R_{ij}h_{j}(T_{0j}-T_{0i})\delta_{i}$$

Equation (15) can be interpreted as a reliable and broad representation of heat transfer through conduction, based on the Onsager–Fuoss model Equation (14), specifically for isothermal multi-component diffusion. An important benefit of utilizing Equation (15) further is its simplicity, as it does not include second order derivatives. By directly comparing Equation (14) with Equation (15), it becomes evident that the overall heat transfer coefficients (**h**) in Equation (14) behave similarly to concentration, but the reference velocity (v_i_) is substituted with the constant reference temperature (T_0j_). Nevertheless, the relaxation term is not included on the left side of Equation (15) as it is combined with the overall heat transfer coefficient. From a theoretical point of view, Equation (14) allows a detailed stability analysis following previous work for isothermal multi-component diffusion [44,45] and by replacing the chemical potential gradient by the term $\left(\frac{\partial T}{\partial x_{i}}\frac{1}{T^{2}}\right)$ as well as molar concentrations by overall heat transfer coefficients (*h_j_*).

The above Equation (15) could be further applied to derive an alternative framework for heat transfer as shown in the results and discussion section.

## 3. Results and Discussion

**Figure 1:**
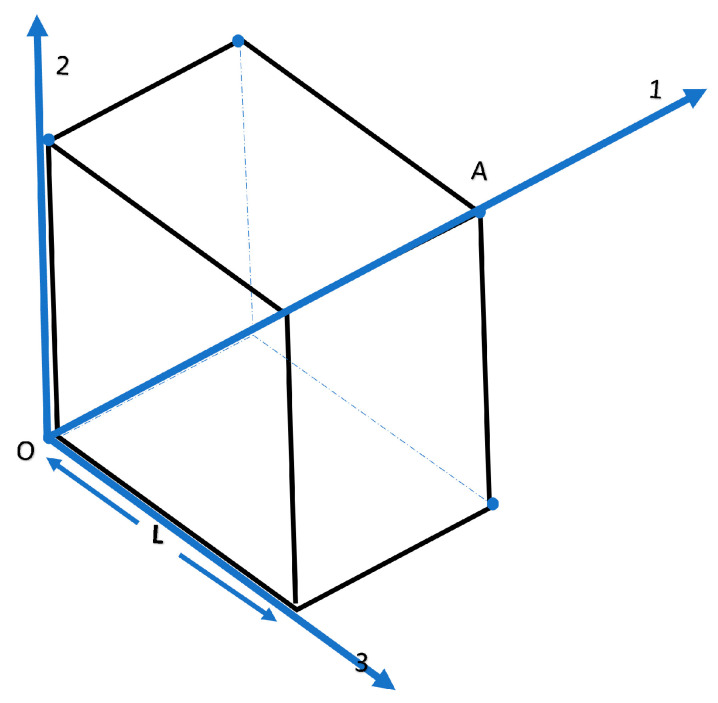
Schematic representation of heat transfer in a cube.

In order to further validate the theory, one has to resort to computation experimentation. In our numerical experiment, heat transfer in an anisotropic cube (see Figure 1), made of wood or a composite material, having edge equal to 1 m. was considered. To introduce the LEIT, we assume an appropriate initial disturbance, such as a sinusoidal starting disturbance of temperature at a point of the cube [14]. In this experiment, the conditions for point A (Figure 1) as summarized in Table 1, were assumed. The reference temperatures were located outside the cube at planes 12, 13 and 23, respectively. Our task is to calculate the resistance coefficients for heat transfer.

The resulting value of T_03_ by using Equation (6) is 293.75 K.

By further using the definition of local heat transfer coefficients Equation (4) as well as the film theory ($h_{loc,ij}=K_{ij}/L_{i}$) Equation (15) is rewritten as:

(16) $$\begin{array}{c} h_{loc,ij}\left(\frac{T-T_{0i}}{K_{ij}}\right)=T^{2}\sum_{j=1}^{3}R_{ij}h_{j}(T_{0j}-T_{0i})\;\mathrm{or}\;\\ T=T_{0i}+L_{i}T^{2}\sum_{j=1}^{3}R_{ij}h_{j}(T_{0j}-T_{0i}) \end{array}$$

The above equation is rewritten by introducing the modified resistance coefficients ($R_{ij}^{*}=R_{ij}T^{2}$) as:

(17) $$\begin{array}{c} h_{loc,ij}\left(\frac{T-T_{0i}}{K_{ij}}\right)=T^{2}\sum_{j=1}^{3}R_{ij}h_{j}(T_{0j}-T_{0i})\;\mathrm{or}\;\\ T=T_{0i}+L_{i}\sum_{j=1}^{3}R_{ij}^{*}h_{j}(T_{0j}-T_{0i}) \end{array}$$

By introducing in Equation (17) the definition of overall heat transfer coefficients Equation (5) the following equation is directly derived:

(18) $$J_{qi}=h_{i}L_{i}\sum_{j=1}^{3}R_{ij}^{*}h_{j}(T_{0j}-T_{0i})\delta_{i}$$

The data summarized in Table 1 were introduced in Equations (17) and (18) by further using the ORR and the theoretical framework of previous section. The resulting nonlinear algebraic system was solved by utilizing standard methods of numerical analysis [46]. The resulting values for the modified resistance coefficients ($R_{ij}^{*}$) are summarized in Table 2.

Using Equation (12), the rest resistance coefficients $R_{ii}^{*}$ could be computed directly from the data reported in Table 2. Regarding stability considerations, one could directly show by using the above calculated resistance coefficients, the heat fluxes illustrated in Table 1 and Equation (1) that σ = 3.66 × 10^−2^ W.m^−3^K^−1^ > 0.

Given the resistance coefficients and the heat transfer coefficients (see Table 2), we could directly calculate $\partial T/\partial x_{i}$ *i = 1*, *2*, *3* by further using the Onsager–Fuoss model for heat transmission (Equation (15)). By using these data, one could also directly estimate thermal conductivity and relaxation time tensors from the Maxwell-Cattaneo model Equation (2) by repeating this process at different times. This necessity arises from the fact that we have to calculate the derivative of heat flux with respect to time in order to apply the Maxwell-Cattaneo model Equation (2).

In order to better validate the work’s findings, let us modify the reference temperatures, T_0i_, by adding a constant temperature T_0_. It is directly demonstrated that the temperature T is increased by the same temperature T_0_ by assuming that the resistance coefficients stay nearly constant for slight temperature variations and by accounting for Equation (6): $\sum_{i=1}^{3}w_{i}=1$; $\sum_{i=1}^{3}w_{i}T_{0i}=T$.

On the other hand, one could expect that the heat fluxes stay unchanged. The resistance coefficients displayed also in Table 2 are the result of a computing experiment that used the remainder of the data described in Table 1 and assumed a fluctuation of both temperature T and reference temperatures by 10 K. This may be explained by the fact that, in accordance with Equations (17) and (18), temperature and fluxes depend only on the difference in the reference temperature (T_0i_–T_0j_).

There is a clear physical meaning to the above computational experiment: If a measurement apparatus located outside the heat transfer framework in Euclidean space is re-calibrated by measuring an additional constant temperature T_0_, an observer of heat transfer located outside the heat transfer framework will observe a different temperature T + T_0_ while the fluxes and dissipation function stay unchanged.

However, the above assumptions might hold true only for Euclidean space. In a relativistic framework one could have to take into account the variation in certain quantities such as mass or energy between the observer and the body. Another major assumption in the Euclidean space is that the resistance coefficients do not depend on the framework. Please note that work in the field of heat conduction in a relativistic extended framework has appeared in the literature [47]. The authors aim to generate interest among peers in the field to extend these ideas to the relativistic context, where non-equilibrium thermodynamics plays a crucial role in numerous cosmological models.

## 4. Conclusions

This study re-evaluates the suitability of the Onsager reciprocal relation (ORR) for describing heat conduction in an anisotropic substance, such as a crystal that is much removed from a state of equilibrium. Research has demonstrated that the Onsager reciprocal relation (ORR) in LEIT has been successfully validated by utilizing heat transfer coefficients. This validation is based on the assumption that the resistance coefficients, entropy production rate, and heat fluxes are uniquely defined and independent of the constant reference temperature. Furthermore, a new equation for heat transmission at the limit of LEIT, similar to the Onsager–Fuoss model used for isothermal multi-component diffusion, was developed. This work could be used for educational purposes and to further understand heat transfer in anisotropic bodies at the limit of LEIT.

## Figures and Tables

**Table 1:** Input data in numerical experiment.

Physical Quantity	Value	Units
T	290	K
J_q1_	0.53	W.m^−2^
J_q2_	0.6	W.m^−2^
J_q3_	−0.51	W.m^−2^
T_01_	270	K
T_02_	280	K
w_1_	0.1	dimensionless
w_2_	0.1	dimensionless
w_3_	0.8	dimensionless

**Table 2:** Output data of the numerical experiment.

Physical Quantity	Value	Units
$R_{12}^{*}$	0.234	K.m. W^−1^
$R_{13}^{*}$	−0.03322	K.m. W^−1^
$R_{23}^{*}$	0.0434	K.m. W^−1^
*h*_1_	0.0265	Wm^−2^K^−1^
*h*_2_	0.06	Wm^−2^K^−1^
*h*_3_	0.136	Wm^−2^K^−1^

## Data Availability

The data of this work is available upon request.

## Nomenclature

*a*_di_	auxiliary parameter of the system
c_i_	molar concentration
**h**	overall heat transfer coefficient
**h_loc_**	close to equilibrium local heat transfer coefficients
**h_res_**	residual heat transfer coefficients
$J_{i}^{*}$	molar flux of the i-th substance relative to the velocity of the centre of mass
**J_q_**	heat flux
**K**	thermal conductivity coefficient
*L*_i_	distance between the point where *T*_0j_ is located and the point having
	temperature *T*.
**R**	resistance coefficients
T	absolute temperature
T_0_	reference absolute temperature
t	time
v**_i_**	velocity of the i-th substance
x_j_	space coordinate
**X**	thermodynamic driving force
**w**	weighting factors
Greek Letters	
**δ**	unit vector
λ_i_	auxiliary parameter
$\mu_{i}$	chemical potential of the i-th substance
σ	entropy production rate per unit volume
Τ	relaxation time.
Subscripts	
d	diffusion
